# Multiple Multilateral Coronary-Cameral Fistulae in a Patient with Minor Cardiac Venous System

**DOI:** 10.1155/2014/754703

**Published:** 2014-02-06

**Authors:** Darko Markota, Zrinko Prskalo, Ivica Markota, Boris Starcevic, Josip Maskovic, Monika Tomic, Ivica Brizic

**Affiliations:** ^1^Department of Cardiology, University Hospital Mostar, Bijeli Brijeg bb, 88000 Mostar, Bosnia and Herzegovina; ^2^Department of Cardiology, University Hospital Dubrava, Avenija Gojka Šuška 6, 10 000 Zagreb, Croatia; ^3^Department of Radiology, University Hospital Mostar, Bijeli Brijeg bb, 88000 Mostar, Bosnia and Herzegovina

## Abstract

A 40-year-old man was hospitalized in the coronary care unit with chest pain and abnormal electrocardiogram. Twenty days earlier, the patient underwent laparoscopic gallbladder surgery. Due to chest pain and ischemic ECG changes, patient was subjected to coronary angiography. The selective coronary angiography revealed multiple multilateral fistulae arising from the left anterior descending artery, circumflex artery, and the right coronary artery draining to the left ventricle. Multislice computed tomography showed hypoplastic coronary sinus and minor cardiac venous system.

## 1. Introduction

Coronary-cameral fistulae are rare congenital or acquired malformation with a direct connection between the coronary arteries and heart chambers. The incidence of coronary artery ventricular fistulae was about 0.1% of congenital cardiac malformation and patients undergoing the coronary angiography [1, 2]. Most of them arise from the right coronary artery and drainage to the right side of the heart [3]. Multiple coronary artery fistulae originating from both the left and right coronary arteries are very rare [4]. Coronary-cameral fistulae mostly present as angina pectoris, syncope, myocardial infarction, heart failure, and cardiac arrhythmia.

## 2. Case Report

**Figure 1:**
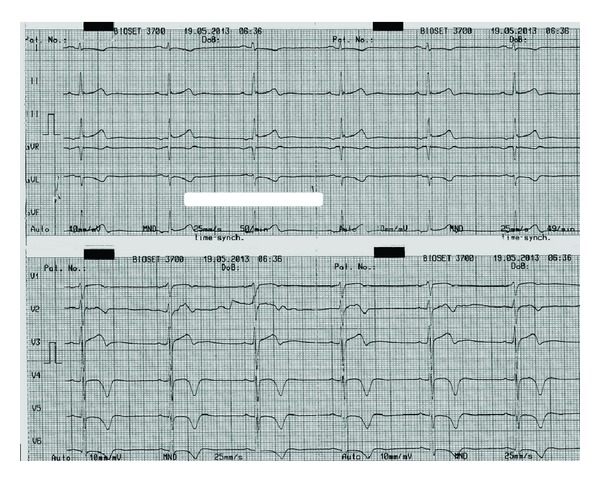
The electrocardiogram at the admission.

**Figure 2:**
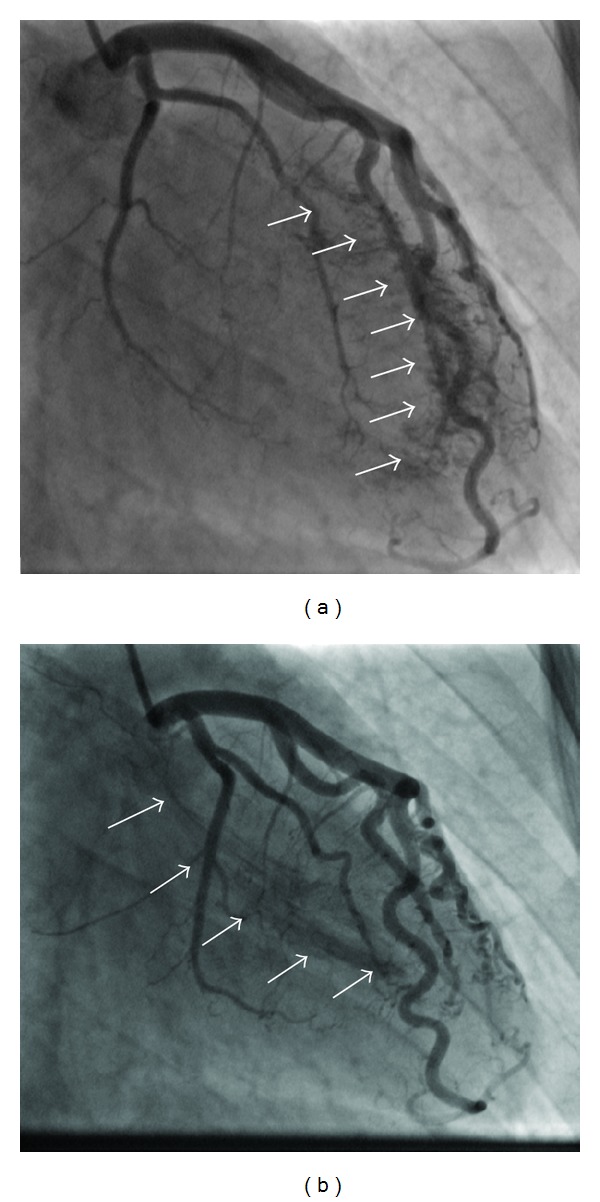
((a), (b)) Selective coronary angiography shows multiple fistulae from the distal left anterior descending coronary artery and diagonal braches to the left ventricle (arrows).

**Figure 3:**
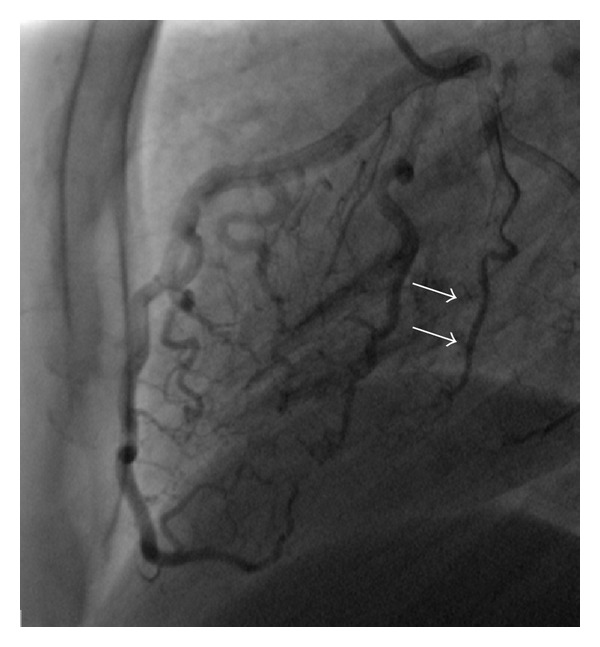
Selective coronary angiography shows multiple fistulae from the artery circumflex to the left ventricle (arrows).

**Figure 4:**
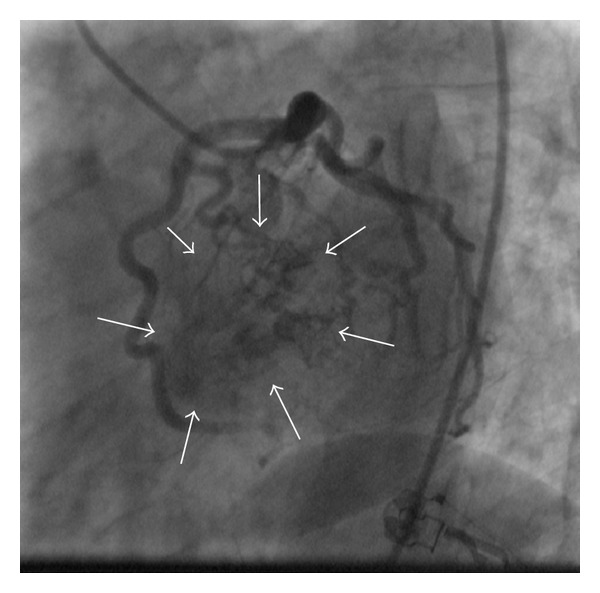
Selective coronary angiography (*spider view*) shows a large mass of contrast in the left ventricle (arrows).

**Figure 5:**
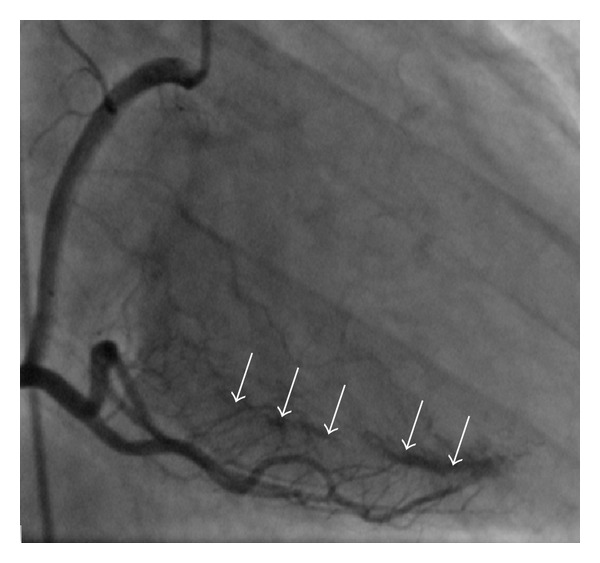
Selective coronary angiography shows multiple fistulae from the middle and distal right coronary artery to the left ventricle (arrows).

**Figure 6:**
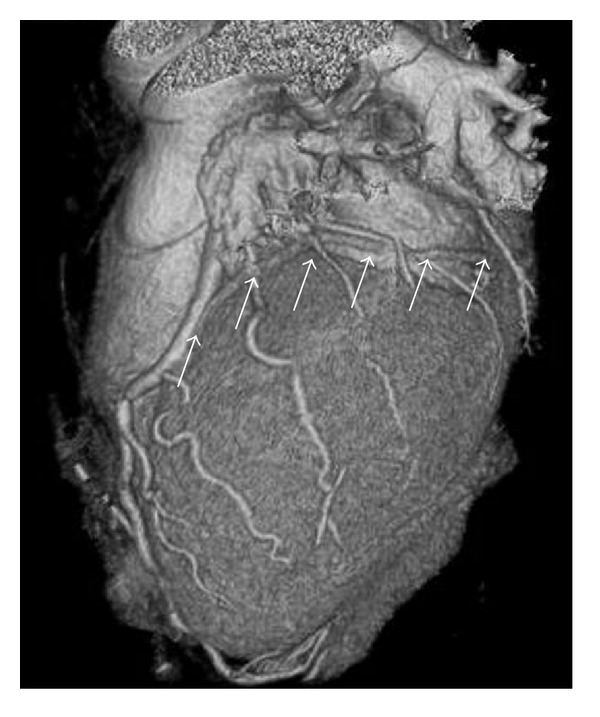
Multislice computed tomography of heart shows minor cardiac venous system (arrows).

A 40-year-old man was hospitalized in the coronary care unit with left sided chest pain and an abnormal electrocardiogram (T wave inversion in precordial leads) (Figure 1). The previous personal and family history of cardiovascular disease was empty. Twenty days prior to the admission, the patient was subjected to laparoscopic gallbladder surgery. The physical examination was normal. Echocardiography did not show any abnormality. All laboratory tests including troponin were within the reference range. Due to chest pain and ischemic ECG changes patient was subjected to invasive cardiology investigation. The selective coronary angiography revealed multilateral multiple fistulae arising from the left anterior descending artery, circumflex artery, and the right coronary artery draining to the left ventricle. There was no significant atherosclerotic lesion in the coronary arteries (Figures 2, 3, 4, and 5). Right heart catheterization showed normal pressure and oxygen partial pressure in the right atrium, right ventricle, and pulmonary arteries. With the right heart catheterization coronary sinus did not show. Multislice computed tomography showed hypoplastic coronary sinus and minor cardiac venous system (Figure 6).

## 3. Discussion

Multiple coronary cameral fistulae terminating in the left ventricle are uncommon, and only a few reports are presented in the literature [5]. Small coronary-cameral fistulae usually did not induce significant cardiac disturbances. Large and multiple coronary-cameral fistulae can induce steal phenomenon, cardiac ischemia, angina pectoris, arrhythmias, and heart failure. The coronary-cameral fistulae are mostly associated with hypertrophic cardiomyopathy or abnormal cardiac vein system [6], as in this case. Cardiac surgery, percutaneous transcatheter embolic occlusion, and conservative therapy are choices in managing coronary fistulae [7, 8]. Due to the minor venous system and multiple fistulae in our patient cardiac surgery and embolisation were not a therapeutic option. Our choice of treatment was bisoprolol, without nitrates and calcium antagonists that can deteriorate steal syndrome. The patient was asymptomatic at the discharge. A control subsequent exercise test did show no abnormality. Over the next six months the patient was hospitalized due to the chest pain twice.

## References

[1] Yamanaka O, Hobbs RE (1990). Coronary artery anomalies in 126,595 patients undergoing coronary arteriography. *Catheterization and Cardiovascular Diagnosis*.

[2] Gillebert C, van Hoof R, van de Werf F, Piessens J, de Geest H (1986). Coronary artery fistulas in an adult population. *European Heart Journal*.

[3] Luo L, Kebede S, Wu S, Stouffer GA (2006). Coronary artery fistulae. *The American Journal of the Medical Sciences*.

[4] Black IW, Loo CK, Allan RM (1991). Multiple coronary artery-left ventricular fistulae: clinical, angiographic, and pathologic findings. *Catheterization and Cardiovascular Diagnosis*.

[5] Arslan S, Gurlertop Y, Elbey MA, Karakelleoglu S (2009). Multiple coronary-cameral fistulae causing angina pectoris. *Texas Heart Institute Journal*.

[6] Ucar O, Cicekcioglu H, Cetin M, Ileri M, Aydogdu S (2011). Coronary artery-left ventricular microfistulae associated with apical hypertrophic cardiomyopathy. *Cardiology Journal*.

[7] Iglesias JF, Thai HT, Kabir T, Roguelov C, Eeckhout E (2010). Transcatheter coil embolization of multiple bilateral congenital coronary artery fistulae. *Journal of Invasive Cardiology*.

[8] Said SA (2011). Current characteristics of congenital coronary artery fistulas in adults: a decade of global experience. *World Journal of Cardiology*.

